# Supplementary material for: Insulin-Degrading Enzyme Is a Non Proteasomal Target of Carfilzomib and Affects the 20S Proteasome Inhibition by the Drug
Source: Biomolecules. 2022 Feb 16;12(2):315. doi: 10.3390/biom12020315 (PMC8869475; doi:10.3390/biom12020315)
Supplement: Supplementary file 1 [file biomolecules-12-00315-s001.zip › biomolecules-1356595-supplementary.pdf]

Figure S1: Testing purify of rIDE and 20S.

rIDE (2 µg) and purified 20S (1 µg) were resolved by SDS-PAGE under denaturing and reducing conditions. Proteins were visualized by Coomassie Brilliant Blau (CBB). rIDE was further probed with the anti-IDE antibody used throughout this study.

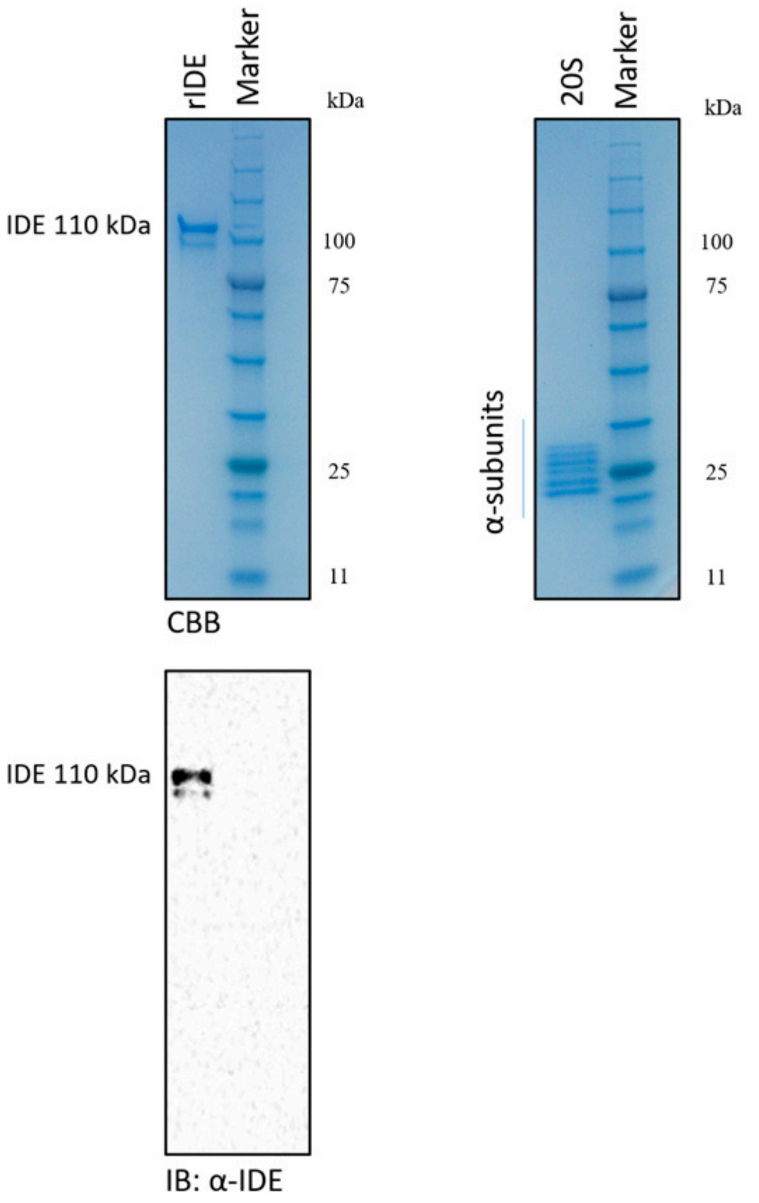

Figure S2: Testing IDE for degradation of CFZ.

Content of CFZ in the samples containing the peptide alone (CFZ) or co-incubated with IDE (CFZ+IDE), Ins (CFZ+Ins) or Ins and IDE (CFZ+Ins+IDE) at 37°C up to 100 min. CFZ is not appreciably degraded by IDE in the presence or in the absence of Ins within the time of the experiment (up to 1.5 h)

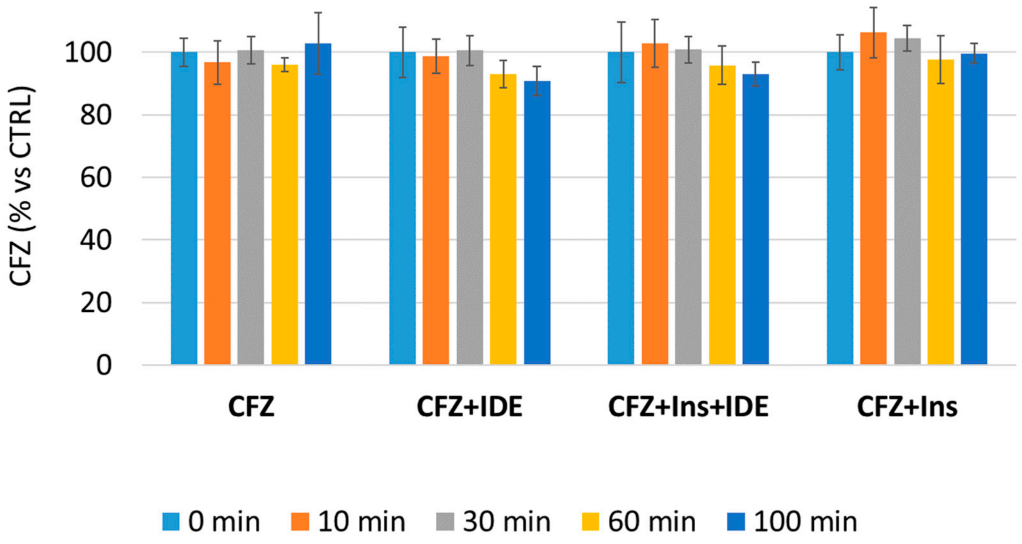

Figure S3: Testing CFZ CC_{50} in rMC1 cells grown in standard cultivation medium.

rMC1 were seeded in 96-well Transwell plates. After an overnight incubation, cell monolayers were stimulated with a wide range of CFZ concentrations. After 24h of stimulation, cell viability was assayed by MTS assay. The CC_{50} was determined by plotting the normalized O.D. calculated at each time point on the log of CFZ concentration. The experiment was run in triplicate (n=3). Data are the Mean±SD. The $LogIC_{50}$±SD and the IC_{50} are reported.

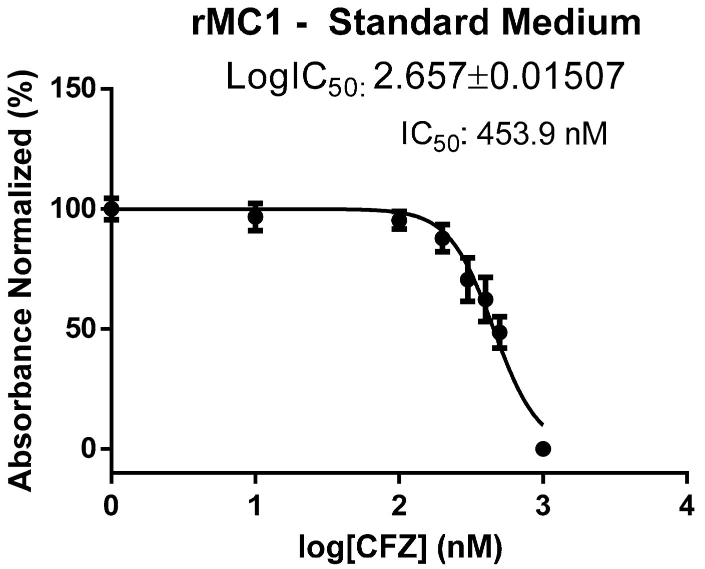

Figure S4: Testing CFZ CC_{50} in rMC1 cells grown in standard cultivation medium.

(A) rMC1 were stimulated with 50 nM 75 nM and 100 nM siRNA targeting IDE or 50 nM non-targeting pool of oligonucleotides (Pool) (limitedly to the figure show). As internal control, cells were stimulated with siRNA vehicle (Ctrl). As expected, the very high dilution of Vehicle (1:2000) did not affect cell behavior (*data not shown*) and is fully comparable to untreated cells. After 72h of incubation cell lysates were immunoblotted (IB) for IDE and β-actin, used as internal control. Histograms report the Mean±SD of the intensity calculated for IDE immunostaining normalized on that of actin. The experiment was run in triplicate (n=3). One-way ANOVA followed by Tukey's post-hoc significance test, ***p<0.0001;

(B) Determination of CFZ CC_{50} of rMC1 grown in OptiMEM in the presence and absence of 50 nM IDE siRNA or non-targeting Pool. Data (Mean±SD) were run in triplicate (n=3) and O.D. for each experimental condition plotted on the log of CFZ concentration. The $LogIC_{50}$±SD and the IC_{50} are reported.

A

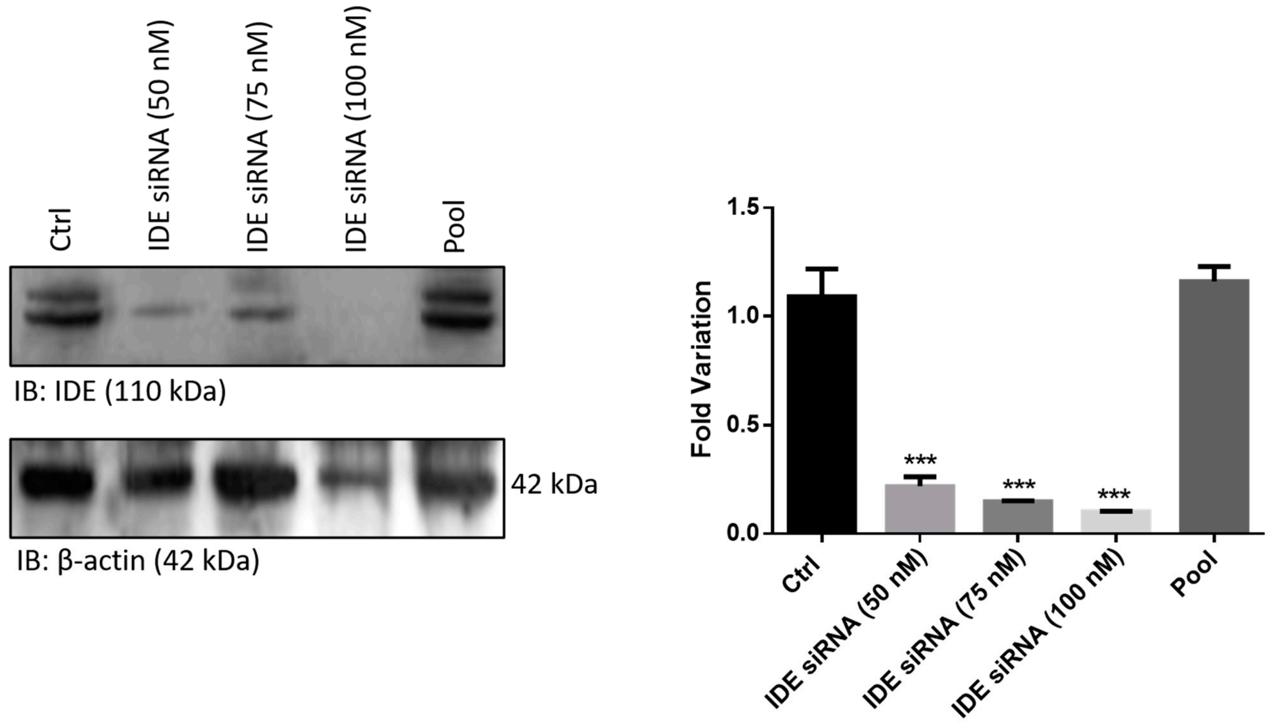

B

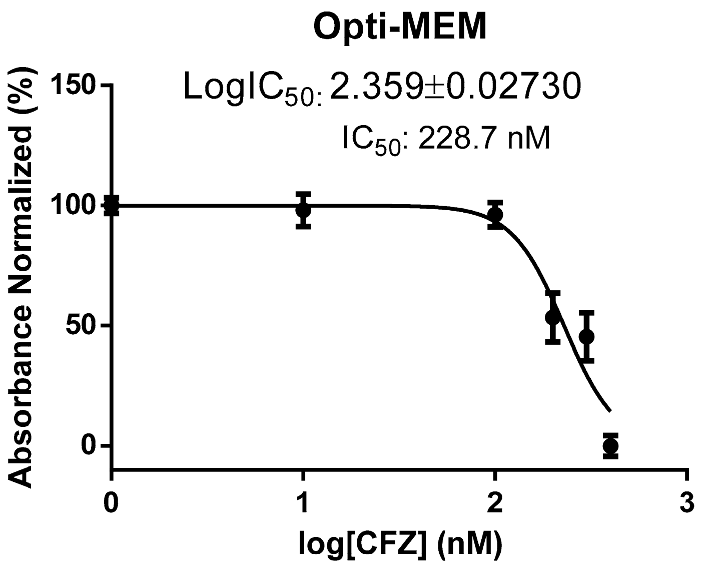

Opti-MEM
LogIC50: 2.359±0.02730
IC50: 228.7 nM
Absorbance Normalized (%)
150
100
50
0
0
1
2
3
log[CFZ] (nM)

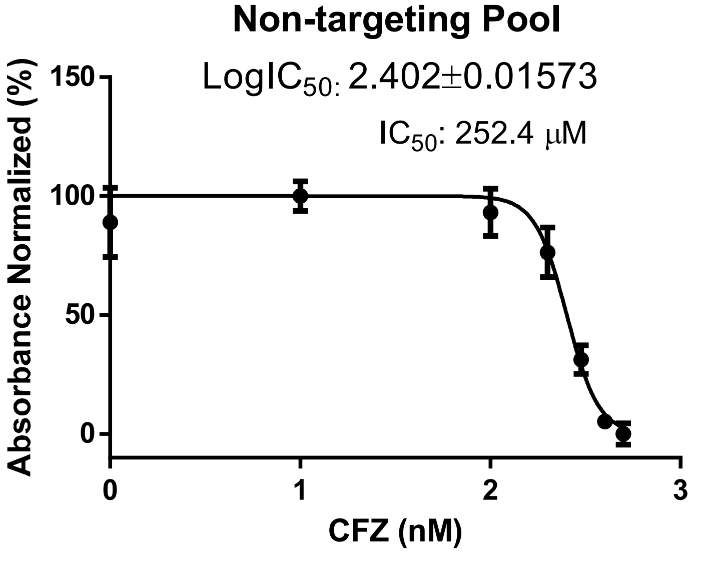

Non-targeting Pool
LogIC50: 2.402±0.01573
IC50: 252.4 μM
Absorbance Normalized (%)
150
100
50
0
0
1
2
3
CFZ (nM)

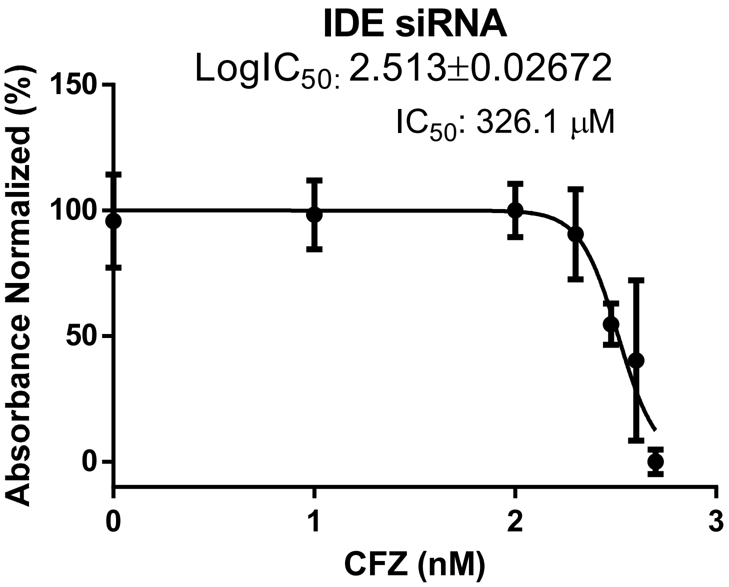

IDE siRNA
LogIC50: 2.513±0.02672
IC50: 326.1 μM
Absorbance Normalized (%)
150
100
50
0
0
1
2
3
CFZ (nM)
